# Model-based intensification of CHO cell cultures: One-step strategy from fed-batch to perfusion

**DOI:** 10.3389/fbioe.2022.948905

**Published:** 2022-08-22

**Authors:** Anne Richelle, Brandon Corbett, Piyush Agarwal, Anton Vernersson, Johan Trygg, Chris McCready

**Affiliations:** ^1^ Sartorius Corporate Research, Brussels, Belgium; ^2^ Sartorius Corporate Research, Toronto, ON, Canada; ^3^ Sartorius Corporate Research, Umeå, Sweden

**Keywords:** bioprocess, mathematical modeling, CHO cells, upstream, intensification, perfusion, fed-batch

## Abstract

There is a growing interest in continuous processing of the biopharmaceutical industry. However, the technology transfer from traditional batch-based processes is considered a challenge as protocol and tools still remain to be established for their usage at the manufacturing scale. Here, we present a model-based approach to design optimized perfusion cultures of Chinese Hamster Ovary cells using only the knowledge captured during small-scale fed-batch experiments. The novelty of the proposed model lies in the simplicity of its structure. Thanks to the introduction of a new catch-all variable representing a bulk of by-products secreted by the cells during their cultivation, the model was able to successfully predict cellular behavior under different operating modes without changes in its formalism. To our knowledge, this is the first experimentally validated model capable, with a single set of parameters, to capture culture dynamic under different operating modes and at different scales.

## 1 Introduction

Currently, only one out of every 10,000 new drug candidates reaches the market. It takes on average 10 years from the discovery of a drug compound until its approval by federal agencies. The probability of clinical success is less than 10% (from Phase one to launch) (Dowden and Munro, 2019; Berdigaliyev and Aljofan, 2020). As a consequence, the cost of drug development is constantly increasing, with a current annual expenditure of more than 2 billion euros, while the actual revenues do not follow the same trend (DiMasi et al., 2016; Wouters et al., 2020).

In this context, we observe that biopharmaceutical companies tend to outsource their early activities in order to reduce their costs and to be more agile around potential market disruption (Mallapaty, 2017; Landhuis, 2018). This implies a growing need to accelerate the operational tasks (process development and product manufacturing). The path to acceleration for biopharmaceutical industry relies mostly on digitalization of process information (to fasten process development) and intensification of operations (to increase productivity and enable more flexibility in the production) (Steinwandter et al., 2019; Narayanan et al., 2022).

Digital bioprocessing is expected to provide significant competitive advantage to industry adopters (e.g., rapid process prototyping, improved process performance and product quality, and de-risked transfer to manufacturing) (Portela et al., 2021; Narayanan et al., 2022). This digital transformation requires the computerization of the information used and generated at each step of a product development process. Once all the information is digitized, it needs to be accessible, organized and contextualized (i.e., data management). This structured digital information can therefore be used to feed data analytics and associated modeling tools to generate valuable insights for further process optimization and control, thereby fasten process development (Finelli and Narasimhan, 2020; Banner et al., 2021; Wasalathanthri et al., 2021).

Today, the industry standard for proteins production, such as monoclonal antibodies, is a fed-batch process (Chen et al., 2018; Ha et al., 2022). However, the productivity of such a process can be significantly improved by implementing a continuous culture strategy to intensify the volumetric productivity. Such approach can lead to an increase up to 10-fold of space-time yields, therefore leading to a reduction of production time by 30% (Müller et al., 2022). These improvements enable opportunity for much smaller facilities with similar or larger productivity outputs, limiting the capital investment (for facilities and raw material costs) and providing manufacturing flexibility and sustainability (Bielser et al., 2018; Chen et al., 2018; Müller et al., 2022).

Such transition from traditional (fed)-batch to continuous manufacturing is facilitated by the emergence of various technological enablers (Rathore et al., 2022) and is encouraged by health authorities (i.e., US Food and Drug Administration). However, the adoption is relatively slow as many challenges remain. Indeed, scale-down models, decisional tools, equipment and procedures currently in place, in most companies, have been developed for fed-batch processes and cannot be transposed without significant changes (Chen et al., 2018). Therefore, this transition might be seeming a high cost and time demand investment to modify existing process development protocols (Papathanasiou et al., 2017; Bielser et al., 2018; Müller et al., 2022). In this context, advanced computational tools could be used to elucidate changes in process dynamics and assess the influence of varying operating scenarios. These in-silico tools provide testing platforms for early determination of process bottlenecks at minimum experimental costs and enable the design of advanced optimization strategies that will lead to optimal and stable operation (Papathanasiou et al., 2017; Rathore et al., 2022).

While this burden for digital transition and technology transfer has been observed in the past (and successfully overcome) in other industry sector (e.g., petrochemical companies, aeronautics), biopharma faces the additional challenge of its operation relying on complex biological systems that cannot be easily described with known first principles rules (Strube et al., 2018). Numerous modelling studies successfully characterizing the influence of measurable process conditions on culture dynamic exist in literature (Smiatek et al., 2020; Wang et al., 2020; Tsopanoglou and Jiménez del Val, 2021; Babi et al., 2022; Schwarz et al., 2022). Unfortunately, they often depend on numerous measurements not often available at manufacturing scale and/or complex modeling and optimization procedures requiring important computational expertise. Therefore, these model-based intensification strategies are difficult to transfer at industrial scale in spite of their most likely success (Papathanasiou et al., 2017; Papathanasiou and Kontoravdi, 2020). Here, we focused on the development of a modeling structure enabling the description of upstream bioprocess dynamics and the transfer between operations (specifically, from fed-batch to continuous culture) at different scales (from Ambr® 250 to Univessel® 2L) with a single set of kinetic parameters. We have demonstrated that the growth model identified using fed-batch cell cultures can be used to design intensified culture conditions in a one-step strategy. To our knowledge, this is the first experimentally validated methodology providing simulation capabilities appropriate for optimization and system configuration decisions within biopharmaceutical process development and advanced control activities.

## 2 Materials and methods

### 2.1 Cell line, inoculum development, medium and analytical methods

Chinese Hamster Ovary (CHO) DG44 cell line (Sartorius) expressing a monoclonal antibody (mAb, IgG1) was used. All experiments were carried out using the same chemically defined media (Sartorius) and Stock Culture Medium (SCM) for the seed train. The seed train cultures were performed in 5 steps. For the fed-batch processes, these pre culture steps were performed in (unbaffled) shake flasks. For the perfusion culture, the last pre-culture step was performed in a 2L Univessel®. The first and second pre culture steps were performed in SCM with 15nM MTX while the others were without MTX. Cells were seeded at 0.2 × 10^6^ cells/mL and split every 3–4 days. The incubators settings for the shake flasks were: 7.5% CO_2_, temperature at 36.8°C, 80% humidity, 120 rpm for agitation with an orbital diameter of 50 mm. Fed-batch, intensified and perfusion cultures were performed with a production medium (PM - Sartorius) and with two feed media (FMA and FMB -Sartorius). Cell growth (VCC and viability) were measured using a Flex analyzer (Nova Biomedical).

### 2.2 Fed-batch and intensified cultures in Ambr® 250

The fed-batch (Experiments 1-4) and intensified cultures (Experiment 5-7) were performed in Ambr® 250 bioreactor with a working volume of 200 and 210 ml respectively for fed-batch and intensified cultures. The cultures were inoculated at 0.3 × 10^6^ cells/mL. The feeding profiles were calculated off-line following the standard applications implemented in Sartorius. For the intensified cultures, the flow rate was adjusted daily from day 3. The culture conditions were controlled at 36.8°C for the temperature, pH 7.1 with CO_2_, 60% of DO with O_2_ and air inlets, 855 rpm for the agitation (adjusted during culture to maintain DO at 60%). 30 µL of antifoam (Sigma antifoam C 2%) was automatically added every 12 h and manually added if needed. A daily glucose bolus was performed starting on day 5 of culture if the measured glucose concentration was less than 5 g/L (stock glucose solution of 400 g/L).

### 2.3 Perfusion cultures in 2L bioreactor

The perfusion culture (Experiment 8) was performed in 2L Univessel® bioreactor with a working volume of 200 ml. The culture was inoculated at 0.3 × 10^6^ cells/mL. The perfusion medium was a mix of 91.2% of PM, 8% FMA, 0.8% FMB and 6mM of l-glutamine. The culture conditions were controlled at 36.8°C for the temperature, pH 6.95 ± 0.05 with CO_2_ and 1M NaCO_3_, 60% of DO with O_2_ and air inlets, 260 rpm for the agitation (adjusted during culture to maintain DO at 60%). 1 ml of antifoam (Sigma antifoam C 2%) was automatically added every day and manually added if needed. The flow rates of media additions and outlet of culture medium are detailed in Section 3.3.

## 3 Theory/calculation/modeling/theoretical aspects

### 3.1 Bioprocess description

Chinese Hamster Ovary (CHO) cells are typically cultivated in a bioreactor with controlled environmental conditions. CHO cell culture population can be divided into 3 subgroups: living ( $X_{v}$ ), dead ( $X_{d}$ ) and lysed ( $X_{l}$ ) cells. Cell death and lysis are cellular processes triggered by the accumulation of metabolic byproducts (here represented by a catch-all “biomaterial” variable $\emptyset_{b}$ ).

A bioreactor can be operated in different modes by acting on the inlet (feeding medium addition, $F_{f}$ ) and outlet ( $F_{out}$ ) flow rates:

- Batch mode: all substrates are added at the beginning of the culture and nothing is added ( $F_{f}=0$ ) or removed ( $F_{out}=0$ ) from the bioreactor afterwards;
- Fed-batch mode: the bioreactor is fed continuously in culture medium ( $F_{f}\neq 0$ ), while the outflow remains nul ( $F_{out}=0$ );
- Continuous mode: the bioreactor is fed continuously ( $F_{f}\neq 0$ ) and the culture medium is continuously removed ( $F_{out}\neq 0$ ).

**FIGURE 1:**
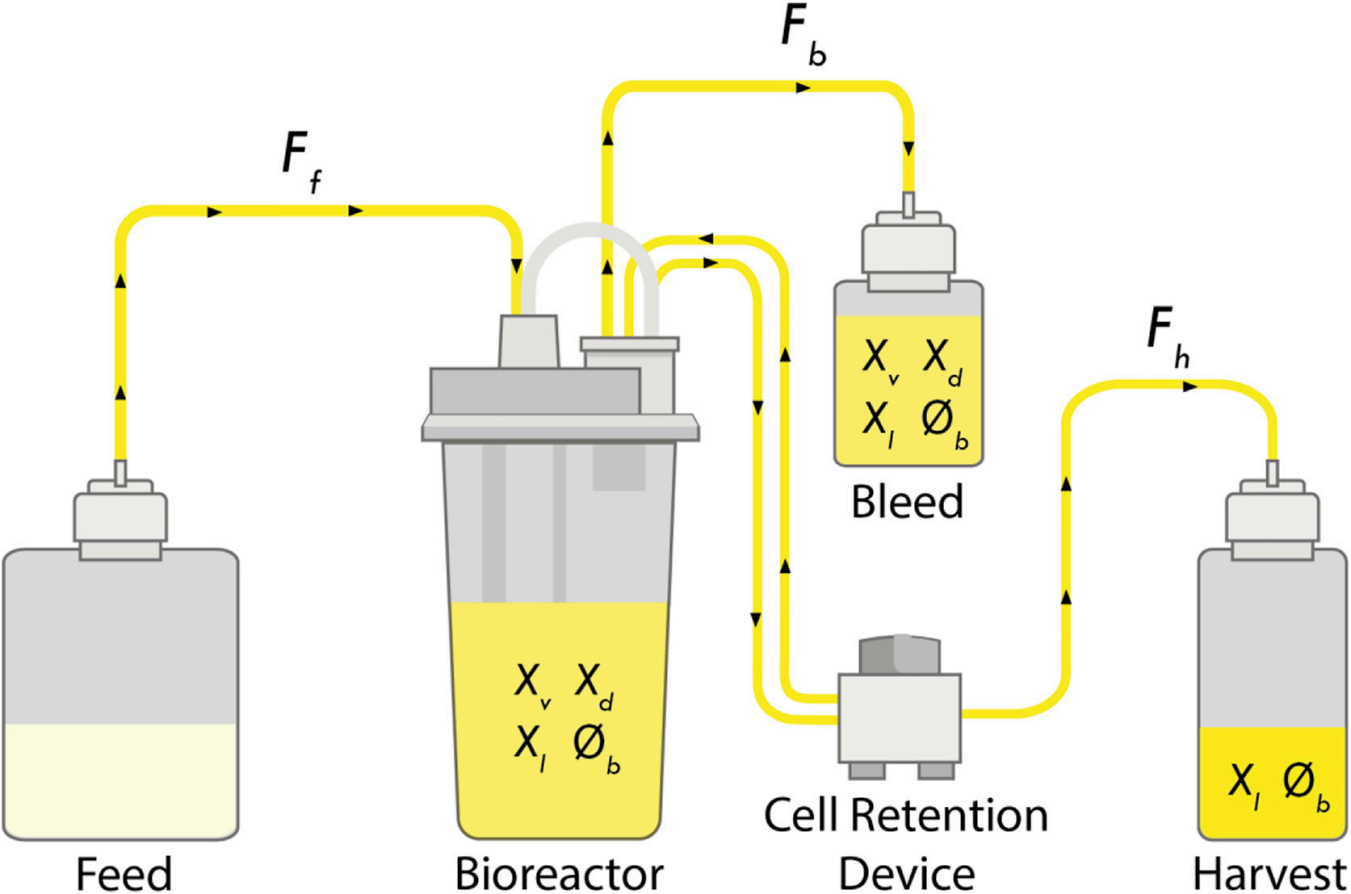
Schematic of a perfusion bioreactor. Media is continuously fed into the bioreactor ( $F_{f}$ ) and a cell-free harvest is continuously removed ( $F_{h}$ ). The cell retention filter is assumed to be ideal, where only the lysed cells ( $X_{l}$ ) and other cellular by-products ( $\emptyset_{b}$ ) pass through while viable ( $X_{v}$ ) and dead cells ( $X_{d}$ ) are fed back into the bioreactor. The bleed stream ( $F_{b}$ ), containing same content as the bioreactor, is used to keep the concentration of living cells within the bioreactor constant, by removing cells in excess.

Perfusion culture is a type of continuous operation where the viable cell concentration ( $X_{v}$ ) and the volume ( V ) within the bioreactor are kept constant (Figure 1). Specifically, the cell culture is continuously fed with fresh medium ( $F_{f}$ ) while the outlet flow ( $F_{out}$ ) is composed of a harvest ( $F_{h}$ ) and a “bleed” ( $F_{b}$ ) streams that are removed to, respectively, keep the culture volume and viable cell concentration constants. To this end, the harvest stream ( $F_{h}$ ) is firstly directed through a cell retention device that will separate the living ( $X_{v}$ ) and dead ( $X_{d}$ ) cells from the used media containing lysed cells ( $X_{l}$ ) and metabolic by-products ( $\emptyset_{b}$ ). The living ( $X_{v}$ ) and dead ( $X_{d}$ ) cells are then re-injected in the bioreactor while the cell-free stream is collected for further purification of the drug product. The harvest flow rate ( $F_{h}$ ) is controlled to keep the volume within the bioreactor constant. The “bleed” outflow ( $F_{b}$ ), presenting the same composition as the bioreactor, is used to maintain the culture at steady-state (i.e., maintain the concentration of living cells within the bioreactor constant) (Bielser et al., 2018; Bausch et al., 2019).

### 3.2 Model development

The general dynamic of a bioprocess can be described by expressing the mass balance of each component ( $\omega_{i}$ ) of the system. In the case where the cell culture take place in a perfectly mixed liquid phase, mass balance performed on the term $V\omega_{i}$ describes the evolution of the component $\omega_{i}$ (expressed as total amount in the bioreactor) over the bioprocess and is defined with the following differential equation:

$$\frac{d\left(V.\omega_{i}\right)}{dt}=\;\underset{k∼i}{\sum }\left(\pm \right)\varphi_{k}+F_{f}\omega_{i,in}-F_{out}\omega_{i}$$ (1)

where the notation k∼i means that the summation is made on all reactions k which imply the component i and $\varphi_{k}$ is the rate of the reaction k . $F_{f}$ and $F_{out}$ represent respectively the inlet feeding and the outlet rates. $\omega_{i}$ and $\omega_{i,in}$ represent respectively the concentration of the component i in the bioreactor and in the feeding.

Using this formalism, the dynamic of the 3 cell population subgroups ( $X_{v}$ , $X_{d}$ and $X_{l}$ ) and the bulk set of metabolites secreted by the cells ( $\emptyset_{b}$ ) can be described with the following set of ordinary differential equations (Eqs 2–5):

$$\frac{d\left(V.X_{v}\right)}{dt}=\mu_{eff}X_{v}V-\mu_{d}X_{v}V-F_{b}X_{v}$$ (2)

$$\frac{d\left(V.X_{d}\right)}{dt}=\mu_{d}X_{v}V-k_{l}X_{d}V-F_{b}X_{d}$$ (3)

$$\frac{d\left(V.X_{l}\right)}{dt}=k_{l}X_{d}V-F_{h}X_{l}-F_{b}X_{l}$$ (4)

$$\frac{d\left(V.\emptyset_{b}\right)}{dt}=X_{v}V-F_{h}\emptyset_{b}-F_{b}\emptyset_{b}$$ (5)

where $X_{v}$ is the viable cell density (VCD—concentration of living cells), $X_{d}$ is the dead cell density (concentration of dead cells), $X_{l}$ is the lysed cell density (concentration of lysed cells), and $\emptyset_{b}\;$ is a catch-all “biomaterial” variable representing the metabolic byproducts. $F_{b}$ is the bleeding rate, $F_{h}$ is the harvest rate, and V is the bioreactor volume. $\mu_{eff}$ , $\mu_{d}$ , and $k_{l}$ are the effective growth, effective death, and lysing rates, respectively.

Here, we assumed that lysed cells $X_{l}$ are a degradation product of the dead cells $X_{d}$ while these arise from living cells $X_{v}$ , according to the specific death rate $\mu_{d}$ . Kroll et al. (Kroll et al., 2016) showed that the lysed cells could be a direct degradation product of living cells $X_{v}$ . However, we were not able, in the case of this study, to discriminate the dead and lysed cells with the analytical assays used to assess the cell viability (trypan blue exclusion test (Strober, 2015)). Therefore, we decided to keep the lysed cell subpopulation as a degree of freedom in the model structure.

As the term $V\omega_{i}$ represents the total amount of component i in the bioreactor, the mass balance equations can be transformed to be expressed with concentration units by combining with Eqs 6, 7:

$$\frac{d\left(V.\omega_{i}\right)}{dt}=V\frac{d\left(\omega_{i}\right)}{dt}+\omega_{i}\frac{d\left(V\right)}{dt}$$ (6)

$$\frac{d\left(V\right)}{dt}=F_{f}-F_{h}-F_{b}$$ (7)

Doing so, the Eqs 2–5 can be rewritten as follows:

$$\frac{dX_{v}}{dt}=\left(\mu_{eff}-\mu_{d}-\frac{F_{f}}{V}+\frac{F_{h}}{V}\right)X_{v}$$ (8)

$$\frac{dX_{d}}{dt}=\mu_{d}X_{v}-\left(k_{l}+\frac{F_{f}}{V}-\frac{F_{h}}{V}\right)X_{d}$$ (9)

$$\frac{dX_{l}}{dt}=k_{l}X_{d}-\frac{F_{f}}{V}X_{l}$$ (10)

$$\frac{d\emptyset_{b}}{dt}=X_{v}-\frac{F_{f}}{V}\emptyset_{b}$$ (11)

These mass balance equations are developed under the assumption that the bleed stream ( $F_{b}$ ) has the same content as the bioreactor and the harvest stream ( $F_{h}$ ) is cell free (ideal separation filter where the lysed cells and biomaterial pass through, and only viable and dead cells are retained into the bioreactor) (Figure 1).

The cell growth rate ( $\mu_{eff}$ ) is represented as the product of the maximal growth rate ( $\mu_{max}$ ) and a nonlinear factor that describes the inhibition of growth due to the accumulation of byproducts (represented by the biomaterial variable $\emptyset_{b}$ ). The resulting effective growth rate is captured in the following equation:

$$\mu_{eff}=\mu_{max}\frac{1}{{\left(\frac{\emptyset_{b}}{K_{I,\emptyset_{b}}}\right)}^{3}+1}$$ (12)

where $K_{I,\emptyset_{b}}$ is a parameter that represents the concentration of biomaterial ( $\emptyset_{b}$ ) above which inhibition occurs (Supplementary Figure S1).

Note that the description of the cell growth rate ( $\mu_{eff}$ ) can be generalized to consider additional effects such as activation by substrates or quadratic effects due to variations in the process environmental conditions (e.g., temperature and pH shifts):

$$\mu_{eff}=\mu_{max}.\overset{S}{\underset{i=1}{\prod }}\eta_{S,i}.\overset{Q}{\underset{i=1}{\prod }}\eta_{Q,i}.\overset{I}{\underset{i=1}{\prod }}\eta_{I,i}$$ (13)

where $\eta_{s,i}$ is the contribution for the substrate variable $S_{i}$ , $\eta_{Q,i}$ is the contribution for the quadratic variable $Q_{I}$ and $\eta_{I,i}$ is the contribution for the inhibitor variable $I_{i}$ . The choice of the mathematical formalism used to describe each of these contributions depends on the case under study. Here, there was no modification of the process conditions nor substrates limiting the cell culture metabolism. Therefore, the formalism used in Eq. 12 was sufficient to describe the effective growth rate observed in the experiments.

The effective death rate, $\mu_{d}$ , is dependent on a base death rate and a toxicity factor related to the accumulation of lysed cells ( $X_{l})$ . Functionally:

$$\mu_{d}=k_{d}+k_{t}X_{l}$$ (14)

where $k_{d}$ is the primary death rate and $k_{t}$ represents the toxicity factor associated to the accumulation of lysed cells in the bioreactor. Note that the toxicity factor could also be related to the accumulation of the metabolite byproducts ( $\emptyset_{b}$ ) in the culture medium. We tested both implementations and obtained better results using the lysed cells ( $X_{l})$ as toxicity factor (data not shown).

Finally, the lysing process was assumed to be governed by $k_{l}$ through a first-order rate law. This assumption was not further investigated as the lysed cell subpopulation is acting as a degree of freedom in the model structure. Tracking the material balance of viable and dead cells gives an indication of total cells generated, and by extension the number of cells that have lysed and are no longer detectable.

Dead cells amount is evaluated indirectly through cell viability measurement which captures the ratio between viable cells and total cells:

$$Viab=\frac{X_{v}}{X_{v}+X_{d}}\;$$ (15)

### 3.3 Perfusion operations

The rate at which the media is exchanged in perfusion culture can be defined either by the cell specific perfusion rate ( CSPR - media supply needed by cells by day) or by the perfusion rate ( P - amount of bioreactor volume renewed by day). Specifically, the cell specific perfusion rate ( CSPR ) is defined as the ratio between the perfusion rate ( P ) and the viable cell density ( $X_{v}$ ) while the perfusion rate ( P ) is defined as the ratio between the feeding rate ( $F_{f}$ ) and the volume of the bioreactor ( V ):

$$CSPR=\frac{P}{X_{v}}$$ (16)

$$P=\frac{F_{f}}{V}$$ (17)

Note that contrarily to the perfusion rate, the CSPR is cell and media specific. Therefore, it represents an important performance criterion (Bielser et al., 2018; Bausch et al., 2019).

Typically, a perfusion culture is set in two phases: intensification and steady-state. Firstly, the cells are growing exponentially until a pre-defined target viable cell concentration ( $X_{v,target}$ ) is reached. During this intensification phase, the feeding rate $(F_{f}$ ) is equal to the harvest rate $(F_{h}$ ) while the bleed stream $(F_{b}$ ) is set to zero. Using Eqs 16, 17, the optimal feeding rate for a given perfusion rate can be defined as follows:

$$F_{f,t_{i}}=\;CSPR.\;X_{v,t_{i}}.V_{t_{i}}$$ (18)

where $F_{f,t_{i}}$ , $X_{v,t_{i}}$ , $V_{t_{i}}$ are respectively the feeding rate, the viable cell concentration, and the bioreactor volume at time i .

The second part of the perfusion culture is called the steady-state phase. It aims to maintain the viable cell concentration in the bioreactor at a predefined target value ( $X_{v,target}$ ) by acting on the bleed rate ( $F_{b}$ ). For this phase, the definition of the feeding rate (Eq. 18) can be simplified as $X_{v,t_{i}}=X_{v,target}$ .

$$F_{f,t_{i}}=\;P.\;V_{t_{i}}$$ (19)

During the steady-state phase, the bleed stream is used as a manipulated process variable to control the viable cell concentration in the bioreactor. In the context of this study, we used a Proportional-Integral (PI) controller to define the bleed rate.

$$F_{b,t_{i}}=\;max\left(0,min\left(F_{f},\;F_{b,t_{i-1}}+\delta_{bleed,t_{i}}\right)\right)$$ (20)

$$\delta_{bleed,t_{i}}=K_{P}.\varepsilon_{bleed,t_{i}}+\frac{K_{P}}{T_{I}}.\left(\varepsilon_{bleed,t_{i}}-\varepsilon_{bleed,t_{i}-1}\right)$$ (21)

$$\varepsilon_{bleed,t_{i}}=X_{v,target}-X_{v,t_{i}}$$ (22)

where $F_{b,t_{i}}$ is the bleed rate at time $t_{i}$ . $\delta_{bleed,t_{i}}$ is the PI controller output (with $K_{P}$ and $T_{I}$ as proportional and integral terms) that will be used to adjust the bleeding rate imposed at time i−1 ( $F_{b,t_{i-1}}$ ) such as to minimize the deviation ( $\varepsilon_{bleed,t_{i}}$ ) of the viable cell concentration at time i ( $X_{v,t_{i}}$ ) from the target setpoint ( $X_{v,target}$ ). For this study, the PI control parameters have been hand tuned and set to $K_{P}=-0.2$ and $T_{I}=0.5$ .

Finally, the harvest rate is determined based on the knowledge of the feed and bleed rates such as to maintain a constant volume:

$$F_{h,t_{i}}=F_{f,t_{i}}-F_{b,t_{i}}$$ (23)

### 3.4 Parameter identification

Dynamic equations were solved by MATLAB’s ordinary differential equation solver function *ode15s*. The parameter identification was performed by using the Nelder–Mead simplex optimization algorithm (function *fminsearch*) in order to minimize a least-squares criterion (sum of squared differences between model predictions and experimental measurements).

$$J\left(\theta \right)=\overset{n}{\underset{j=1}{\sum }}\overset{N}{\underset{i=1}{\sum }}{\left(y_{ij}\left(\theta \right)-y_{mes\_ij}\right)}^{2}$$ (24)

where θ is the vector of the parameters to be identified (dim θ = 5), $\theta^{T}\;$ = [ $\mu_{max}$ $K_{I,\emptyset_{b}}$ $k_{d}$ $k_{t}$ $k_{l}$ ], $y_{ij}^{T}\left(\theta \;\right)$ = [ $X_{v\_ij}$ $Viab_{ij}$ ] is the vector of the simulated variables (using model of mass balance Eqs 8–11) at the *i*th time instant in the *j*th experiment, $y_{mes,ij}^{T}$ = [ $X_{v,mes\_ij}$ $Viab_{mes\_ij}$ ] is the vector of the corresponding measurements.

### 3.5 Parameter sensitivity analysis and predicted model output uncertainty

The analysis of the sensitivity of the model outputs with respect to the parameters was performed as in Richelle et al. (Richelle et al., 2014). To this end, the four state variables ( $X_{v}$ , $X_{d}$ , $X_{l}$ and $\emptyset_{b}$ ) were defined as the system outputs $y_{j}$ with *i* = 1:4. The parameters were denoted $\theta_{j}$ with *j* = 1:5. The time evolution of the 4 × 5 sensitivity functions ( $\frac{\partial y_{i}}{\partial \theta_{j}}$ ) was then computed as follows:

$$\frac{d}{dt}\left(\frac{\partial y_{i}}{\partial \theta_{j}}\right)=\frac{\partial }{\partial \theta_{j}}\left(\frac{dy_{i}}{dt}\right)=\frac{\partial f_{i}}{\partial \theta_{j}}+\;\overset{m}{\underset{k=1}{\sum }}\frac{\partial f_{i}}{\partial y_{k}}\;\times \frac{\partial y_{k}}{\partial \theta_{j}}$$ (25)

for *i* = 1 to 4, *j* = 1 to 4 and *m* = dim(*y*) = 4 with *dy*_*i*_ */dt = f*_*i*_ (*y, q*_*j*_ *, t*) represented by model Eqs 8–11.

These sensitivity functions were used for computing a lower bound of the variance (Cramer-Rao bound) of the parameter estimation errors ( $\sigma_{\theta_{i}}^{2}$ , *i* =1:5) on the basis of the Fischer information matrix:

$$F=\overset{n}{\underset{j=1}{\sum }}\overset{N}{\underset{i=1}{\sum }}{\left(\frac{\partial y_{ij}}{\partial \theta }\right)}^{T}Q_{ij}^{-1}\left(\frac{\partial y_{ij}}{\partial \theta }\right)$$ (26)

$\sigma_{\theta_{i}}^{2}=S_{ij}$ with $S=F^{-1}$ where $y_{ij}$ = [ $X_{v,ij}$ $X_{d,ij}$ $X_{l,ij}$ $\emptyset_{b,ij}$ ] at the *i*th time instant in the *j*th experiment and $\theta^{T}\;$ = [ $\mu_{max}$ $K_{I,\emptyset_{b}}$ $k_{d}$ $k_{t}$ $k_{l}$ ].

The covariance matrix S could also be used to measure the correlation between the parameters (linear dependencies):

$$COR\left(\theta_{i},\theta_{j}\right)=\frac{S_{ij}}{\sqrt{S_{ii}}\sqrt{S_{jj}}}$$ (27)

where $S_{ij}$ is the covariance of the errors on parameter estimates $\theta_{i}$ and $\theta_{j}$ ; $S_{ii}$ and $S_{jj}$ are respectively the variance of the errors on parameter estimates $\theta_{i}$ and $\theta_{j}$ .

For analysing the uncertainty on the model outputs with respect to the parameter estimation errors, a global approach based on Monte Carlo sampling method was used. Contrarily to local approach based on first-order Taylor series approximation, this approach does not assume that the model responds linearly to a perturbation evaluated at a specific point of the parameter space. Instead, this sampling-based method uses a repeated random sampling of parameter values in a defined parameter space. In doing so, the overall model is used to generate the associated predicted model outputs by an iterative process of model simulations.

## 4 Results

### 4.1 Model identification using fed-batch cultures

**FIGURE 2:**
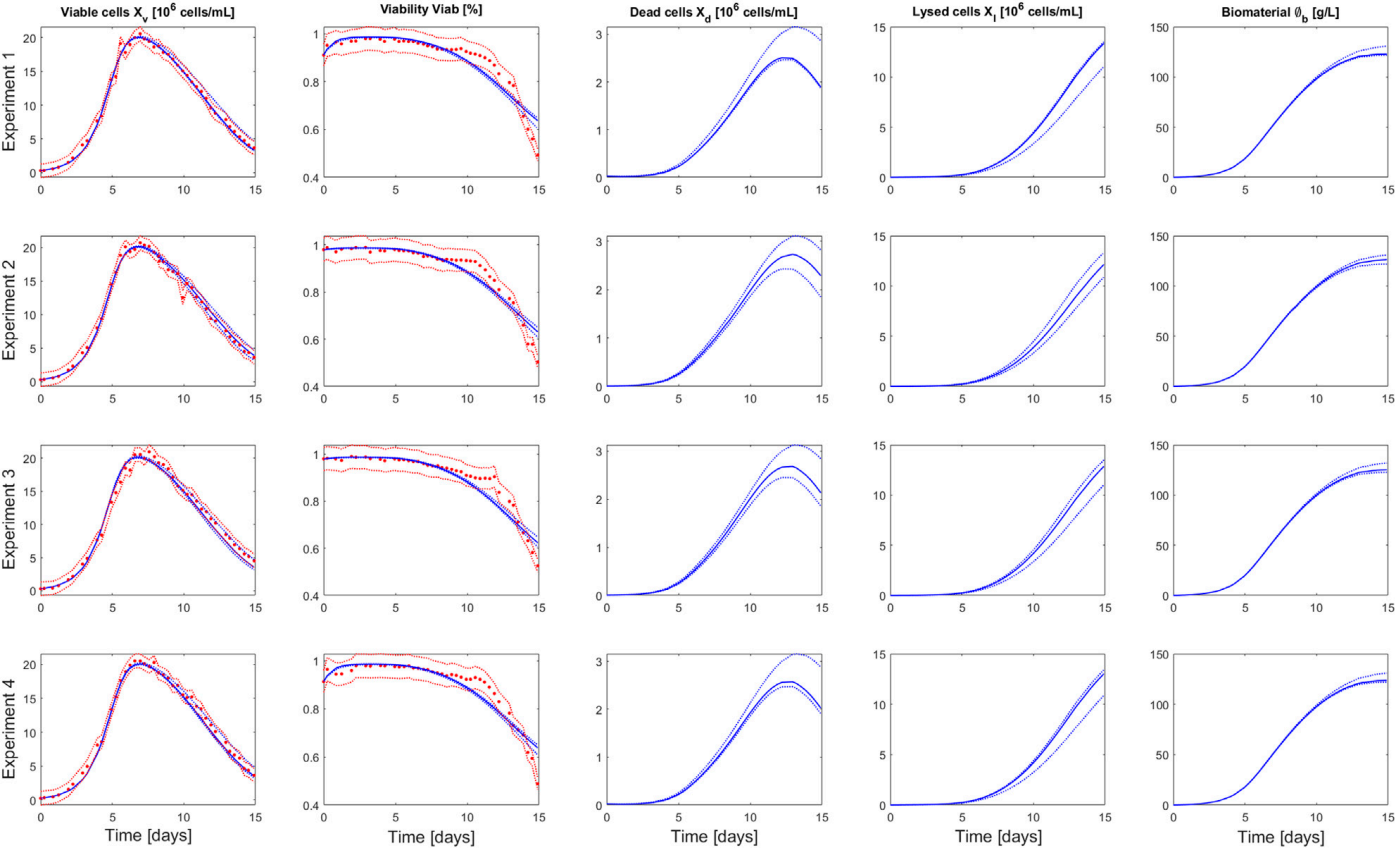
Comparison between measurements of Ambr® 250 fed-batch experiments 1–4 (red dots) and the model simulation (blue curve) performed using the parameters value identified on the whole set of experiments. The dashed red lines represent the experimental confidence interval. The dashed blue lines represent the uncertainty in the model predictions—calculated using Monte Carlo simulations (1000 samples) of normally distributed pseudo random parameters values (parameter space defined by $\theta \pm 2\sigma_{\theta }$ ).

We developed a growth model that tracks density and viability of a cell culture population (living, dead and lysed). The parameters of this model were identified based on 4 replicate fed-batch experiments performed in Ambr® 250 (see Section 2 for details). To circumvent local minima and convergence problems with the optimization algorithm, a multi-start strategy was considered for the initialization of the parameter values. 100 uniformly distributed pseudo-random values over a given range (Table 1) were used for the initialization of the algorithm. For analysing the uncertainty on the model outputs with respect to the parameter estimation errors, a global approach with a Monte Carlo simulation was used, based on 1000 normally distributed pseudo-random sets of parameter values (Figure 2).

**TABLE 1:** Parameters values identified for each experiment separately and whole set of experiments 1 to 4.

	Initialization range	Exp. 1	Exp. 2	Exp. 3	Exp. 4	Exp. 1-2-3-4	$\sigma_{\theta }$ ^a^	CV^b^
$\mu_{max}$	[0.01, 1]	0.8409	0.8560	0.8145	0.8439	0.8384	0.0005	0.06
$k_{d}$	[0.01, 1]	0.0210	0.0275	0.0273	0.0136	0.0209	0.0002	0.87
$k_{t}$	[0.01, 1]	0.0286	0.0261	0.0232	0.0376	0.0290	0.0002	0.62
$K_{I,\emptyset_{b}}$	[1, 100]	24.1117	24.4954	25.9156	23.7059	24.3905	0.0226	0.09
$k_{l}$	[0.01, 1]	0.8723	0.7209	0.8765	0.6352	0.7743	0.0702	9.06

a Standard deviation of parameter values identified using the whole set of experiments.

b Coefficient of variation (CV) of parameter values ( $\sigma_{\theta }/\theta$ - expressed in %) identified on the whole set of experiments 1 to 4.

The identified parameter values (based on the 4 experiments) and associated confidence intervals are presented in Table 1 and the correlation matrix (absolute values of the correlation coefficients between parameters) in Table 2. Results obtained for the parameter identification of each experiment separately are also presented in Supplementary Tables S1–S6. The model simulations and associated prediction confidence intervals are presented in Figure 2 and Supplementary Figure S2 along with the experimental data used to identify the model.

**TABLE 2:** Correlation matrix (absolute value) of the parameters identified on the whole set of experiment.

	$\mu_{max}$	$k_{d}$	$k_{t}$	$K_{I,{Ø}_{b}}$	$k_{l}$
$\mu_{max}$	1	0.1669	0.0626	0.6263	0.0415
$k_{d}$	0.1669	1	0.7480	0.0731	0.1358
$k_{t}$	0.0626	0.7480	1	0.0884	0.4355
$K_{I,\emptyset_{b}}$	0.6263	0.0731	0.0884	1	0.0619
$k_{l}$	0.0415	0.1358	0.4355	0.0619	1

The model captures well the evolution of the viable cell density over the culture duration for the 4 experiments. The viability predictions present larger, but still acceptable, residuals than the ones of the viable cell density. We observe a marked transition in the decrease dynamic of the measured viability, at day 11 of the culture, that the model is not able to capture. Such shift in the viability evolution results, most likely, from a strain-specific adaptation to high level of toxic by-products as it was not systematically observed in cell cultures performed with different strains (data not shown). In this context, we decided to not introduce additional complexity to the model to capture this event.

The parameters were identified with good confidence, this was also reflected in the simulation of the model output uncertainty (Table 1 and Figure 2). The highest parameter correlation is observed between $\mu_{max}$ and $K_{I,\emptyset_{b}}$ followed by $k_{t}$ and $k_{l}$ as expected due to the respective formulation of the effective growth and death rates (Eqs 13, 14). The largest uncertainty was associated to the lysed cells with the parameter $k_{l}$ . This is explained by the fact that lysed cells act as a degree of freedom in the model since no experimental measurements were available for this variable. The determination of living and dead cell density is typically performed with image-based cell analyzer using colorimetric test (dye exclusion methods (Strober, 2015)). However, the lysis of cells is neglected in these analytical assays, leading to false estimation of the dead cell population (Kroll et al., 2016). Some protocols using death markers (lactate deshydrogenase and double stranded genomic DNA) in the culture supernatants for monitoring cell lysis have shown promising results to solve this issue (Klein et al., 2015).

### 4.2 Model-based prediction of intensified operations performance

**FIGURE 3:**
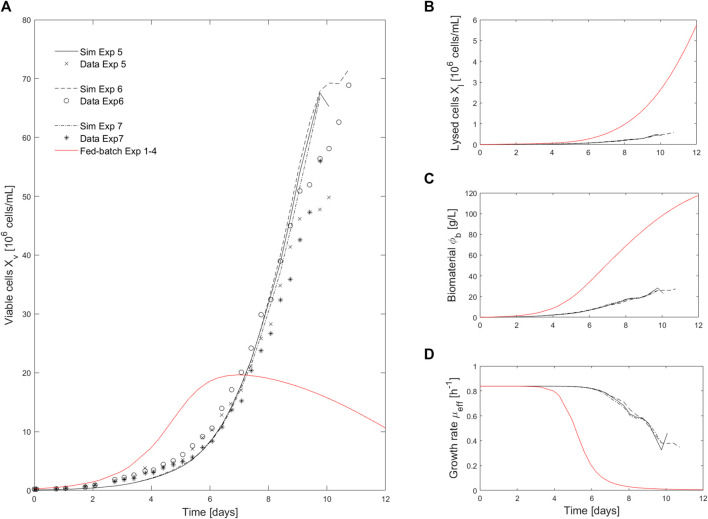
Comparison of intensified (media exchange) and fed-batch experiments. **(A)**. Comparison of viable cell density ( $X_{v}$ ) measurement of intensified cultures 5-7 (black cross, star and open circle) with the model simulation of the intensified culture (solid black line) and the fed-batch culture (solid red-line) performed using the parameters value identified on the whole set of fed-batch experiments 1-4. **(B–D)** present, respectively, a comparison of the lysed cells density ( $X_{l}$ ), biomaterial concentration ( $\emptyset_{b}$ ) and growth rate ( $\mu_{eff}$ ) simulated for the intensified culture (solid black line) and the fed-batch culture (solid red-line) using the parameters value identified on the whole set of fed-batch experiments 1–4.

The model was further cross-validated using data from Ambr® 250 intensified cultures (Experiments 5, 6 and 7 - see Methods for details). The model simulations successfully predicted the culture dynamic for intensified operations with media exchange. The intensification was achieved by harvesting the culture media at the same rate ( $F_{h}$ ) as the medium feeding ( $F_{f}$ ); keeping the living ( $X_{v}$ ) and dead cells ( $X_{d}$ ) into the bioreactor while lysed cells ( $X_{l}$ ) and secreted byproducts ( $\emptyset_{b}$ ) were filtered out (Figure 1). Doing so, cell growth is no longer inhibited by the biomaterial accumulation and the death rate is less favored by the lysed cell accumulation in the culture media (Eq. 14). Specifically, the concentration of lysed cells and biomaterials after 10 days of culture in intensified conditions were respectively 10- and 4-fold lower than for fed-batch operations (Figure 3). Note that we did not present the evolution of the cell viability for these intensified experiments as the measurements and associated simulations present a variation over the culture time smaller than the standard error assumed for this signal (measurements and simulations between 0.99 and 0.97 while the error is assumed to be 5%).

The proposed model has a rather simple structure compared to the ones presented in literature. The overall formalism to describe the different cell population subgroups is, most often, conserved across existing models: cell growth and mortality occurs in parallel while dead cells are lysed over time. The main difference in our proposed structure lies in the description of the growth and dead rates. Indeed, it is well known that mammalian cell metabolism can be limited by the depletion of nutrients or by the accumulation of inhibitory metabolites (Lourenço da Silva et al., 1996). Therefore, the death and growth rate are typically described as extended Monod’s law (more than one compound influencing the reaction rate) accounting for diverse activating and inhibiting compounds.

For example, Shirahata et al. (Shirahata et al., 2019) modelled the growth rate in continuous operation with an inhibition by the accumulation of ammonia. Lourenço da Silva et al. (Lourenço da Silva et al., 1996) developed a kinetic model that describes the growth of hybridoma cells in fed-batch culture with decreasing and death enhancing effects of glucose, amino-acids, serum and oxygen depletion, on the one hand, and of ammonia and lactate accumulation on the other. Craven et al. (Craven et al., 2013) accounted in their growth model for the activation by substrates (glucose and glutamine) and inhibition by byproducts (lactate and ammonia). Papathanasiou et al. (Papathanasiou et al., 2017) used five metabolites (glucose, glutamine, arginine, aspartate, asparagine) to describe their activation and inhibition influence on growth and death processes.

The evaluation of the respective influence of these potential limiting factors is a difficult task. Several of these factors are often simultaneously limiting; leading to observed diversity in the model formalism for growth and death rates. Furthermore, the description of such activation and inhibition effects by multiple metabolites quickly complicates the model structure. Indeed, with this formalism, these compounds are introduced as state variables in the model and their associated kinetics need to be described. The main novelty of the proposed model is the introduction of a catch-all “biomaterial” variable ( $\emptyset_{b}$ ). This variable captures the inhibition of growth by a bulk of secreted by-products without detailing the identity and contribution of each potential inhibitor. Therefore, it simplifies the model structure (and reduced the number of model parameters) as there is no need to describe the dynamic associated to these compounds.

### 4.3 Design and analysis of perfusion process conditions

The perfusion operation of a CHO cell culture was simulated with the proposed model structure using the following assumptions:

- The culture was performed in a 2L bioreactor Univessel®
- The initial seeding density and viability were set as for the Ambr® 250 fed-batch experiments
- The lysed cells and inhibitory biomaterial were initialized at 0
- There were no considerations for adjustment in growth changes during the simulation
- It was assumed that the media composition and perfusion rate is sufficient for supplying nutrients

Different events for process operation changes were introduced to test the capabilities of the model and the cell’s response to switch in operations (Table 3):

- The culture began with an intensification phase to reach the cell density target ( $X_{v,target}$ = 50 × 10^6^ cells/mL). The feed and harvest rate were equal ( $F_{f}$ = $F_{h}$ ) and defined as presented in Methods for a perfusion rate ( P ) of 2.25 vol/day. The bleed rate ( $F_{h}$ ) was equal to zero
- Once the cell density reached 95% of the desired target $X_{v,target}$ , a PI controller was used for adjusting the bleed rate and maintaining a desired setpoint (details of the PI control setup presented in Section 3.3)
- An increase in the perfusion rate ( P ) was introduced for more than a day to test the PI control (in between 12,9 and 14.1 days) before being set back to its original defined value of 2.25 vol/day
- A decrease of the perfusion rate ( P ) was imposed at 21.1 days to assess the response of the cells to an increase of biomaterial accumulation
- Finally, an increase of the cell density target (to $X_{v,target}$ = 70 × 10^6^ cells/mL) was introduced to evaluate the capacity of the cells to cope with strong switch in operations

**TABLE 3:** Details of switch in process operations for perfusion simulation and experimental run.

Event time (days)	P (Vol/day)	$X_{v,target}$ (10^6^ cells/mL)
0	2.25	50
12.9	3	50
14.1	2.25	50
21.1	1.75	50
24	1.75	70

**FIGURE 4:**
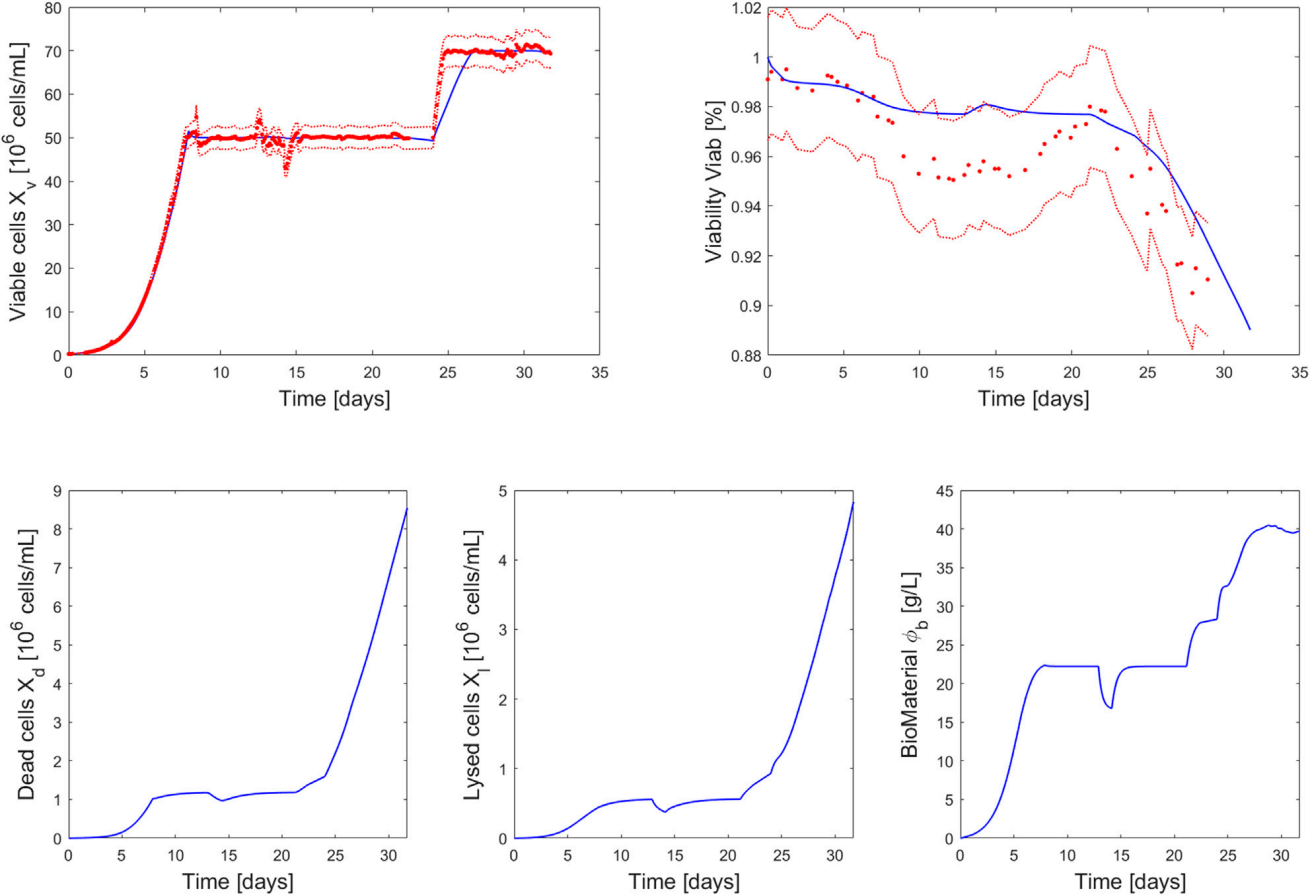
Comparison between measurements of 2L perfusion experiment 8 (red dots closed loop PI control of the viable cell density measured on-line at $X_{v,target}$ setpoint value using $F_{b}$ as manipulated variable) and the model simulation (blue curve—closed loop PI control of the viable cell density predicted by the model at $X_{v,target}$ setpoint value using $F_{b}$ as manipulated variable) performed using the parameter values identified on the whole set of fed-batch experiments 1–4. The dashed red lines represent the experimental confidence interval.

**FIGURE 5:**
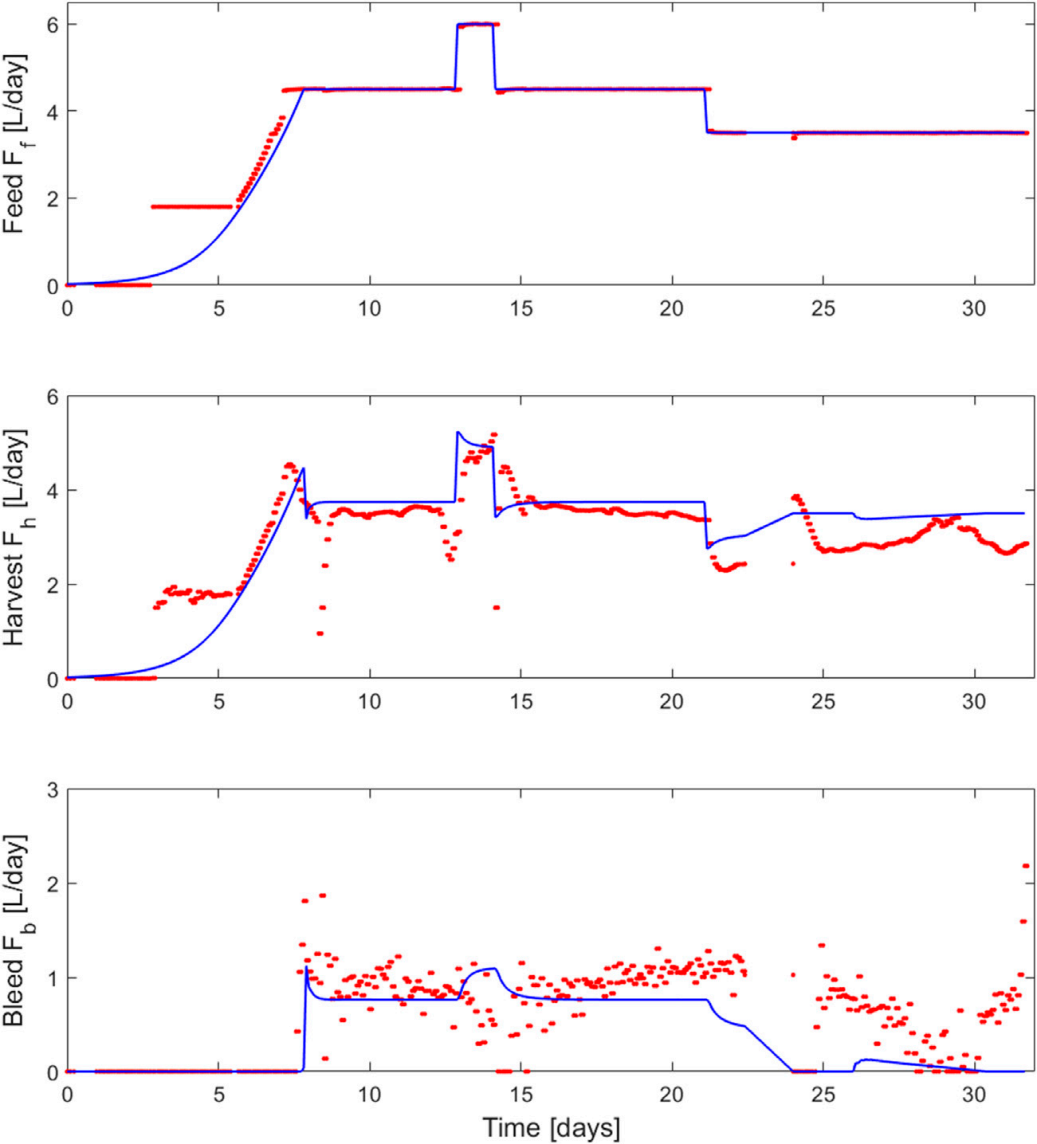
Comparison between the feed ( $F_{h}$ ), harvest ( $F_{h}$ ) and bleed ( $F_{b}$ ) rates implemented during 2L perfusion experiment 8 (red dots—closed loop PI control of the viable cell density measured on-line at $X_{v,target}$ setpoint value using $F_{b}$ as manipulated variable) and the model simulation (blue curve—closed loop PI control of the viable cell density predicted by the model at $X_{v,target}$ setpoint value using $F_{b}$ as manipulated variable) using the parameter values identified on the whole set of fed-batch experiments 1–4.

The simulation of this perfusion experiment is presented in Figure 4 and Figure 5 along with the experimental data collected during a 2L perfusion bioreactor run performed under the same operations (Table 3) and with the same PI controller. Specifically, the PI controller relied on the model prediction of the viable cell density evolution for the simulated run while the viable cell density measured on-line was used when the controller was implemented for the experimental run. The model prediction accurately captured the dynamic of the cell culture in perfusion based on parameter values identified using Ambr® 250 fed-batch experiments. As observed in the model simulations of the fed-batch experiments 1-4 (Figure 2), the viability is associated to larger residuals than the viable cell density, but still holding close to the experimental confidence area. Importantly, the model accurately identified the decrease of cell viability (due to the accumulation of biomaterials) initiated once the perfusion rate was reduced ( P =1.75 vol/day). The model was also able to predict the maximum stable target cell density for the last process operation switch ( $X_{v,target}$ = 70 × 10^6^ cells/mL).

The PI controller adequately adjusted the bleed rate to hold a stable cell density during the simulation and the experimental run. The observed discrepancies between the outflow ( $F_{b}$ and $F_{h}$ ) simulations and the associated stream rates experimentally implemented by the closed-loop PI controller (Figure 5) can be explained by small variations in the on-line viable cell concentration measurements. Also, we encountered some issues with the outflow pumps and associated recording devices during the week-end periods that this experiment covered, as highlighted by the missing experimental points in between days 21 and 24. Such discrepancies could be reduced by further optimizing the controller parameters to ensure a more adequate tuning.

To our knowledge this is the first design of a perfusion culture using a model identified based on fed-batch experiments. Typically, models are developed for one type of culture (batch, fed-batch or continuous) and cannot be transferred to other process operation without changes in the model structure and/or parameter values. Shirahata et al. (Shirahata et al., 2019) modified the formalism of the growth rate function depending on the operation mode. Specifically, in batch mode, they simulated the viable cell dynamic using an activation by glucose and the onset of massive cell death when a glucose depletion occurs. In perfusion mode, the growth rate was no longer modelled in function of substrate consumption but rather with an inhibition due to ammonia accumulation in the culture medium. Lourenço da Silva et al. (Lourenço da Silva et al., 1996) successfully validated a kinetic model for hybridoma fed-batch culture and mentioned that they were capable to simulate experimental results obtained during batch and continuous processes with minor changes of few kinetic parameters. Unfortunately, the data were not shown. Finally, Craven et al. (Craven et al., 2013) developed a unique model structure for CHO cell culture operated under 3 different modes (batch, bolus fed-batch and continuous fed-batch) and grown under 2 scales (3 and 15 L) but the model parameters identified changed with scale and mode of operation.

The presented model-based approach enabled to drastically improves the process performance (Table 4). This represents a reliable alternative to existing experimental procedure such as the ones presented in Janoschek et al. (Janoschek et al., 2018) and Wolf et al. (Wolf et al., 2019). These protocols rely on the evaluation and optimization of different feed, harvest and bleed strategies similar to a Design of Experiments (DoE) approach. While these methods have been proven to be successful, they are experimentally intensive and do not allow the user to test the system response to joint variation of multiple control variables and setpoints.

**TABLE 4:** Volumetric Productivity (VP—10^6^ cells/mL.day) and Space-Time Yield (STY—10^6^ cells/mL.day) associated with the fed-batch (FB), intensified (I) and perfusion (P) experiments at different days of culture (days 7, 10 and 20). Metrics calculated following the standards presented in Bausch et al. (Bausch et al., 2019).

	VP	STY
FB - Exp 1–4 d7	2.3	2.9
I - Exp 5–7 d7	19.3	4.6
I - Exp 5–7 d10	138.7	36.7
P - Exp 8 d7	116.0	30.5
P - Exp 8 d10	112.5	52.6
P - Exp 8 d20	112.5	81.1

## 5 Conclusion

The era of digital transformation has reached biopharma. In this context, in-silico computational tools will be essential to help optimize upstream bioprocesses and accelerate product development and production (Luo et al., 2021).

The goal of this study was to propose a model-based strategy to improve upstream cell culture development within biopharmaceutical manufacturing thanks to its process operation transfer capabilities. Often referred to as in-silico experimentation, subject matter experts (SME) can use the proposed framework to digitally test various hypothetical operating policies. Ideas can be honed and proposed before verifying in the lab. The hypothesized model was built from limited data with a focus on core growth kinetics and sensitivity to biomaterials. It can be used to investigate growth trajectories and evaluate media exchange operating modes (intensified growth and perfusion). To demonstrate these capabilities, the model was calibrated with Ambr® 250 fed-batch experiments and successfully used to forecast growth profiles under various operating modes including the cell line’s response to media exchange. The proposed model-based process design strategy was also tested by collaborators from biopharmaceutical companies with other cell lines producing different products (data not shown). While the set of parameters values identified varied for each case study, the proposed model structure was always capable of predicting changes in culture behavior for different operating conditions, as presented in this study.

As more experiments are run and data is collected, this generic model structure can continuously be extended to include additional metabolic information from shifts in pH, temperature, media composition and other important process conditions. Such model could therefore also be used to optimize media composition and recipe decisions to maximize productivity while maintaining Critical Quality Attributes (CQAs) within specification.

However, models describing the influence of spent media composition on productivity and product quality are far more complex and, currently, not as mature as those for growth description. This relates to growth and death kinetics being driven strongly by the extracellular environment, while productivity and CQAs (e.g., glycan profile) are influenced through more subtle shifts in the intracellular metabolism. For the moment, intracellular measurements are expensive and not practical for typical product development workflows or high throughput experimental designs. Therefore, analytical tool such as machine learning and other data driven methods would most likely be used to relate extracellular process measurements to the CQAs.

To conclude, using simulation is common practice in many process industries but a relatively new tool for biopharmaceutical manufacturing. Being able to test operating strategies digitally reduces wet lab experimental needs, speeding up the product development process. The big picture then - and the takeaway for biopharma companies - is to move toward an enhanced, optimized approach for upstream process development that makes use of existing information to bring transformation, optimization and ultimately, profitability. The key dynamic behind all of it is an integration of advanced data analytics, process knowledge and digital tools that transcend the traditional method of process monitoring and move toward digital twins powered by a systems approach of bio-simulation.

## Data Availability

The original contributions presented in the study are included in the article/Supplementary Material, further inquiries can be directed to the corresponding author.

## Nomenclature

***F*_*b*_**: **L/h** bleed rate
$F_{f}$: **L/h** feeding rate
$F_{h}$: **L/h** harvest rate
$k_{d}$: **1/h** primary death rate
$k_{l}$: **1/h** lysing rate
$k_{t}$: **10** ^**6**^ **cells/h** **.mL** toxicity factor associated to the accumulation of lysed cells
$K_{I,{Ø}_{b}}$: **g/L** biomaterial inhibition parameter of the effective growth rate
P: **vol/day **perfusion rate
${Ø}_{b}$: **g/L** biomaterial concentration
$\mu_{d}$: **1/h** effective death rate
$\mu_{eff}$: **1/h** effective growth rate
$\mu_{max}$: **1/h** maximal growth rate
V: **L** bioreactor volume
Viab: **%** viability
$X_{d}$: **10^6^** **cells/mL** dead cell concentration
$X_{l}$: **10^6^** **cells/mL** lysed cell concentration
$X_{v}$: **10^6^** **cells/mL** viable cell concentration
