# Supplementary material for: Model-based intensification of CHO cell cultures: One-step strategy from fed-batch to perfusion
Source: Front Bioeng Biotechnol. 2022 Aug 22;10:948905. doi: 10.3389/fbioe.2022.948905 (PMC9443430; doi:10.3389/fbioe.2022.948905)
Supplement: Supplementary file 1 [file DataSheet1.docx]

**Supplementary Materials**

**Model-based intensification of CHO cell cultures: one-step strategy from fed-batch to perfusion**

Supp. Table 1 - Standard deviation of parameter value identified for each experiment separately

|  | Exp. 1 | Exp. 2 | Exp. 3 | Exp. 4 |
| --- | --- | --- | --- | --- |
| $\mu_{max}$ | 0.0010 | 0.0010 | 0.0010 | 0.0010 |
| $k_{d}$ | 0.0005 | 0.0004 | 0.0003 | 0.0003 |
| $k_{t}$ | 0.0004 | 0.0003 | 0.0007 | 0.0007 |
| $K_{I,\emptyset_{b}}$ | 0.0459 | 0.0437 | 0.0465 | 0.0444 |
| $k_{l}$ | 0.2132 | 0.3703 | 0.1043 | 0.0670 |

Supp. Table 2 - Coefficient of variation (CV) of parameter values ( $\sigma_{\theta}/\theta$ - expressed in %) identified for each experiment separately

|  | Exp. 1 | Exp. 2 | Exp. 3 | Exp. 4 |
| --- | --- | --- | --- | --- |
| $\mu_{max}$ | 0.12 | 0.12 | 0.13 | 0.12 |
| $k_{d}$ | 2.18 | 1.29 | 2.49 | 2.14 |
| $k_{t}$ | 1.34 | 1.09 | 2.02 | 1.88 |
| $K_{I,\emptyset_{b}}$ | 0.19 | 0.18 | 0.19 | 0.19 |
| $k_{l}$ | 24.44 | 51.36 | 13.43 | 10.55 |

Supp. Table 3 - Correlation matrix (absolute value) of the parameters identified for Experiment 1

|  | $\mu_{max}$ | $k_{d}$ | $k_{t}$ | $K_{I,\emptyset_{b}}$ | $k_{l}$ |
| --- | --- | --- | --- | --- | --- |
| $\mu_{max}$ | 1 | 0.1472 | 0.0678 | 0.6216 | 0.0428 |
| $k_{d}$ | 0.1472 | 1 | 0.8146 | 0.0620 | 0.2018 |
| $k_{t}$ | 0.0678 | 0.8146 | 1 | 0.0874 | 0.2770 |
| $K_{I,\emptyset_{b}}$ | 0.6216 | 0.0620 | 0.0874 | 1 | 0.0626 |
| $k_{l}$ | 0.0428 | 0.2018 | 0.2770 | 0.0626 | 1 |

Supp. Table 4 - Correlation matrix (absolute value) of the parameters identified for Experiment 2

|  | $\mu_{max}$ | $k_{d}$ | $k_{t}$ | $K_{I,\emptyset_{b}}$ | $k_{l}$ |
| --- | --- | --- | --- | --- | --- |
| $\mu_{max}$ | 1 | 0.2089 | 0.0910 | 0.6209 | 0.0424 |
| $k_{d}$ | 0.2089 | 1 | 0.7794 | 0.0960 | 0.1142 |
| $k_{t}$ | 0.0910 | 0.7794 | 1 | 0.1303 | 0.3462 |
| $K_{I,\emptyset_{b}}$ | 0.6209 | 0.0960 | 0.1303 | 1 | 0.0615 |
| $k_{l}$ | 0.0424 | 0.1142 | 0.3462 | 0.0615 | 1 |

Supp. Table 5 – Correlation matrix (absolute value) of the parameters identified for Experiment 3

|  | $\mu_{max}$ | $k_{d}$ | $k_{t}$ | $K_{I,\emptyset_{b}}$ | $k_{l}$ |
| --- | --- | --- | --- | --- | --- |
| $\mu_{max}$ | 1 | 0.1165 | 0.0029 | 0.6560 | 0.0351 |
| $k_{d}$ | 0.1165 | 1 | 0.4454 | 0.0489 | 0.1229 |
| $k_{t}$ | 0.0029 | 0.4454 | 1 | 0.0081 | 0.8097 |
| $K_{I,\emptyset_{b}}$ | 0.6560 | 0.0489 | 0.0081 | 1 | 0.0548 |
| $k_{l}$ | 0.0351 | 0.1229 | 0.8097 | 0.0548 | 1 |

Supp. Table 6 – Correlation matrix (absolute value) of the parameters identified for Experiment 4

|  | $\mu_{max}$ | $k_{d}$ | $k_{t}$ | $K_{I,\emptyset_{b}}$ | $k_{l}$ |
| --- | --- | --- | --- | --- | --- |
| $\mu_{max}$ | 1 | 0.1538 | 0.0076 | 0.6444 | 0.0459 |
| $k_{d}$ | 0.1538 | 1 | 0.4376 | 0.0593 | 0.1163 |
| $k_{t}$ | 0.0076 | 0.4376 | 1 | 0.0046 | 0.8201 |
| $K_{I,\emptyset_{b}}$ | 0.6444 | 0.0593 | 0.0046 | 1 | 0.0675 |
| $k_{l}$ | 0.0459 | 0.1163 | 0.8201 | 0.0675 | 1 |


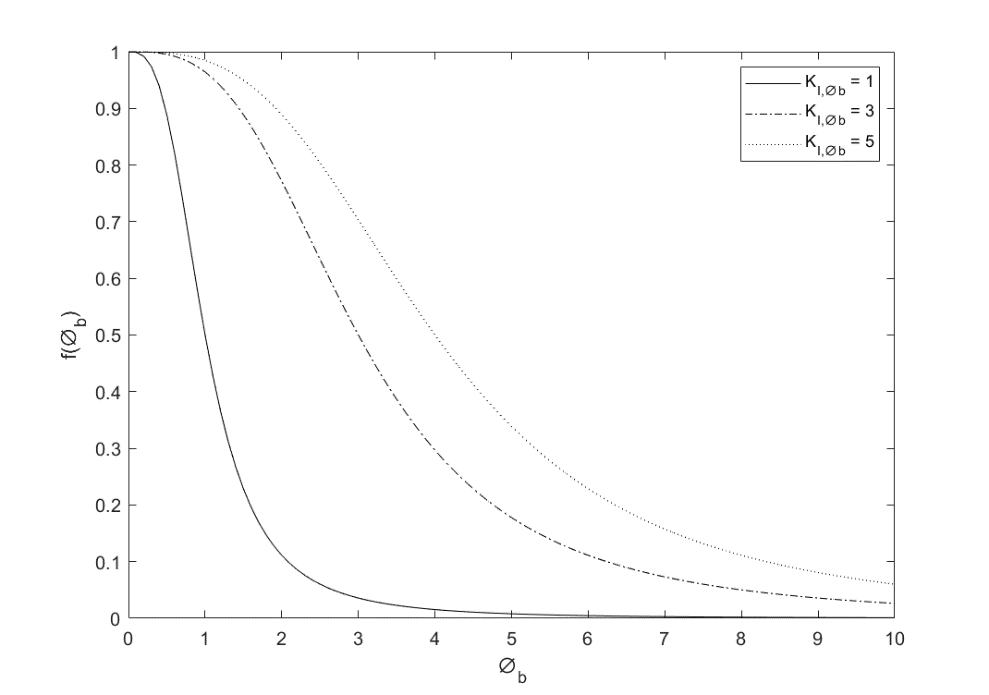


Supp Figure 1 **-** Growth inhibition factor ($f(\emptyset_{b})$) as a function of the biomaterial concentration ($\emptyset_{b})$ value for a range of parameter values $K_{I,\emptyset_{b}}$.


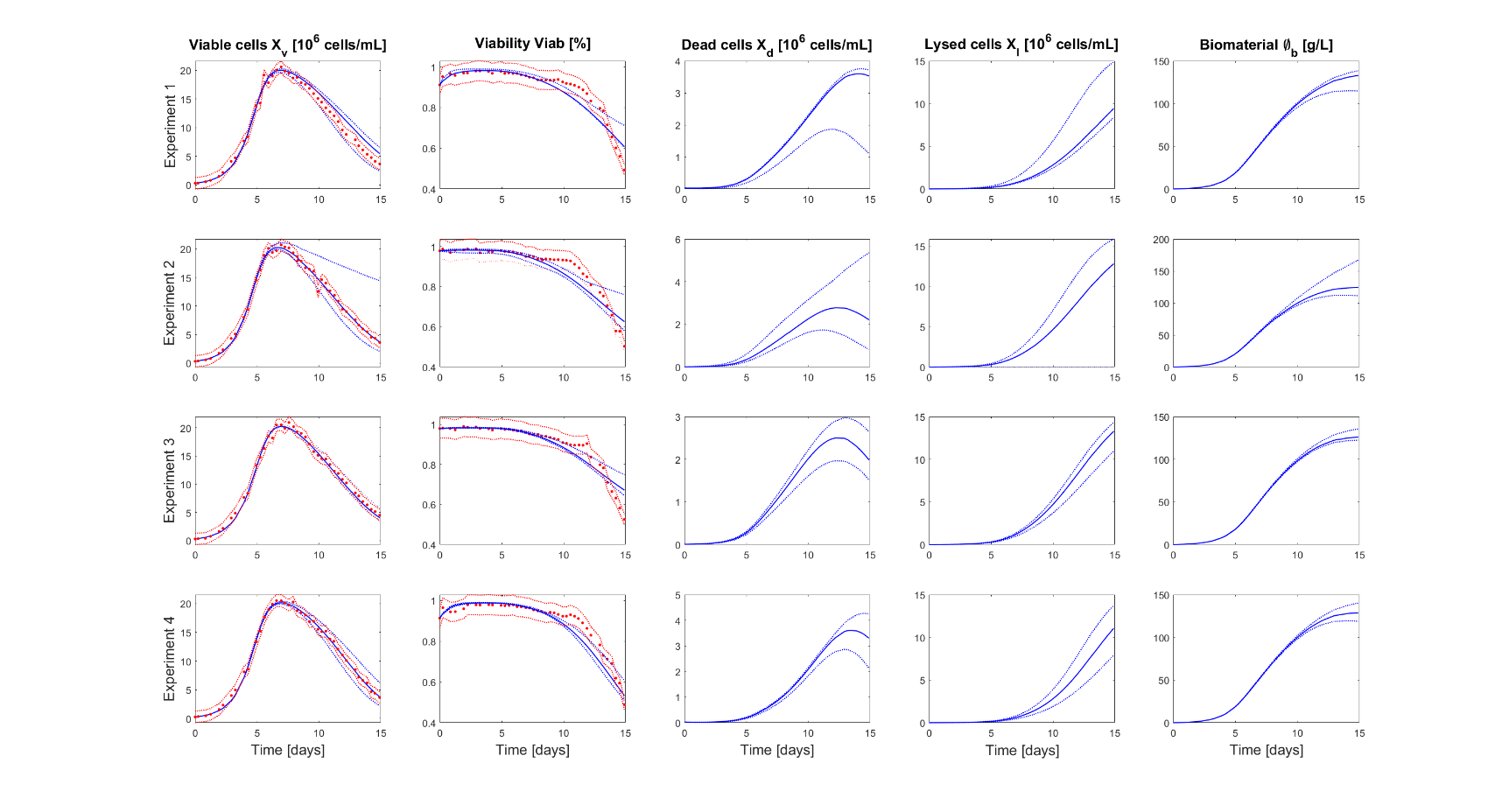


Supp. Figure 2 - Comparison between measurements of Ambr250 fed-batch experiments 1- 4 (red dots) and the model simulation (blue curve) performed using the parameters value identified for each experiment separately. The dashed red lines represent the experimental confidence interval. The dashed blue lines represent the uncertainty in the model predictions – calculated using Monte Carlo simulations (1000 samples) of normally distributed pseudo random parameters values (parameter space defined by $\theta\pm2\sigma_{\theta}$)
